# Evolutionary Diversification and Functional Roles of MYB Transcription Factors in Algae: Insights from Genomic Distribution and Regulatory Networks

**DOI:** 10.3390/biology15201865

**Published:** 2026-10-16

**Authors:** Yurong Wang, Chunli Luo, Xunmeng Li, Yulin Liang, Peipei Xu, Anqi Xue, Hong Du, Jing Chen

**Affiliations:** 1 Guangdong Provincial Key Laboratory of Marine Biotechnology, STU-UNIVPM Joint Algal Research Center, Institute of Marine Sciences, Shantou University, Shantou 515063, China; 24yrwang@stu.edu.cn (Y.W.);; 2 Guangdong Provincial Key Laboratory of Marine Disaster Prediction and Prevention, Shantou University, Shantou 515063, China; 3 Guangdong Engineering Technology Research Center of Offshore Environmental Pollution Control, Shantou University, Shantou 515063, China; 4 National Engineering Research Center for Marine Aquaculture, Zhejiang Ocean University, Zhoushan 316022, China

**Keywords:** MYB transcription factors, algae biotechnology, bibliometric analysis, molecular evolution, phylogeny, regulatory networks, stress response

## Abstract

MYB transcription factors (TFs) represent a conserved family of DNA-binding regulators involved in diverse cellular and physiological processes. MYB TFs are well-characterized in terrestrial plants, but their evolutionary history and functional diversity in algae remain poorly understood. This review summarizes current knowledge of the structural classification, evolutionary characteristics, and regulatory functions of algal MYB TFs. Bibliometric analysis of Web of Science Core Collection publications (1 January 2003–27 April 2026) reveals a marked expansion in algal MYB research since 2019, with emphasis on transcriptional regulation, lipid metabolism, and stress adaptation. Phylogenetic analyses indicate that algal MYBs exhibit lineage-specific diversification, despite retention of MYB domains conserved with land plants. Functional studies further demonstrate that MYB TFs regulate cell cycle progression, carbon-concentrating mechanisms, lipid biosynthesis, nutrient metabolism, and abiotic stress responses. Nevertheless, most algal MYB genes remain functionally uncharacterized. Integrating multi-omics with genome editing will improve understanding of MYB-mediated regulatory networks and facilitate future applications in algal biotechnology and metabolic engineering.

## Simple Summary

Algae support aquatic food webs and can provide materials for food and other useful products. Yet scientists still know little about how algae control their growth and cope with changing conditions. This review brings together studies of a family of proteins that help control which genes are active. We examine how these proteins differ among groups of algae and assess what experiments reveal about their roles. Published studies link some of them to cell division, nutrient use, the production of oils and pigments, and responses to environmental stress. However, most of these proteins have not been tested directly, and differences in how genetic information is analyzed make comparisons between species uncertain. We conclude that these proteins may be important to algal life, but their individual roles require more careful testing. This clearer picture can guide research on aquatic ecosystems and, over time, help develop algae as reliable sources of food, feed, and renewable materials.

## 1. Introduction

MYB transcription factors (TFs) represent one of the largest gene families, acting as key regulators of diverse biological processes [1]. The first MYB gene identified was the *v-myb* oncogene of avian myeloblastosis virus (AMV), cloned by Klempnauer and colleagues in 1982 [2]. The subsequent discovery of its cellular counterpart, the *c-myb* proto-oncogene, led to the identification of a family of transcriptional regulators characterized by a conserved N-terminal DNA-binding domain [3]. In plants, the first *MYB* gene identified was the maize *C1* gene, which shares approximately 40% sequence similarity with animal *MYB* proto-oncoproteins, particularly within the conserved DNA-binding region [4]. While extensively characterized in higher plants, where they regulate development [5], metabolism [6,7], and stress responses [8], their evolution and functional diversification in algae remain poorly understood [9]. As early-diverging photosynthetic organisms, algae are critical for investigating the origin and evolution of plant MYB regulatory networks [10]. 

Algae are a highly diverse group of photosynthetic organisms distributed across marine, freshwater and extreme environments, including both unicellular microalgae and multicellular macroalgae. Because of their ecological importance [11] and biotechnological potential in biofuel production, carbon sequestration and aquaculture, algae have become an important research focus in recent years [12,13,14]. With the rapid development of genome sequencing, transcriptomics and multi-omics technologies, increasing numbers of transcription factor families have been identified in algae [15,16]. Recent bibliometric trends indicate that research on algal MYB TFs has shifted from early gene identification studies toward more integrated analyses of transcriptional regulation, metabolism and stress adaptation. Current studies are mainly concentrated in a limited number of model species, particularly *Chlamydomonas reinhardtii* and *Phaeodactylum tricornutum*. Although publication output has increased substantially in recent years, research in this field is still fragmented.

Structurally, algal MYB proteins show considerable diversity among different lineages. MYB TFs are generally classified into four major groups, including 1R-MYB, 2R-MYB, 3R-MYB and 4R-MYB proteins, based on the number and arrangement of MYB repeats [17]. Among these, 2R-MYBs represent the dominant subgroup in many algae, whereas atypical MYB proteins, such as 5R- and 6R-MYBs, have also been identified in brown algae [18]. Comparative genomic studies revealed substantial variation in MYB abundance across algal taxa, suggesting lineage-specific expansion and diversification of MYB gene families. In particular, green algae and charophyte-related lineages exhibit increasing MYB complexity, indicating progressive diversification of transcriptional regulatory systems during evolution [8,18].

Functional studies have shown that MYB TFs participate in diverse physiological and metabolic processes in algae. In *C. reinhardtii*, MYB proteins regulate cell-cycle progression [19], carbon concentrating mechanisms [20] and nutrient stress responses [21]. In oleaginous microalgae, MYB TFs are closely associated with lipid biosynthesis and triacylglycerol accumulation [22]. MYB regulators have also been implicated in phosphorus and nitrogen limitation, salt stress, light adaptation and carotenoid biosynthesis across multiple algal taxa [23]. Despite these advances, most functional studies remain limited to a few model algae, and the regulatory networks and evolutionary conservation of algal MYB TFs are still poorly understood.

Despite the increasing recognition of MYB transcription factors in algal growth, environmental adaptation, and metabolic regulation, the available evidence remains fragmented across different algal lineages and research topics. Early studies on brown algae have conducted important analyses of the MYB family in brown algae, but this article integrates research from multiple algal lineages, examines research trends, compares MYB structural categories and phylogenetic patterns, and evaluates evidence of cross-species reporting function [18]. A bibliometric overview is included in this review to summarize the development of algal MYB research, identify major research themes, and highlight areas requiring further investigation. This analysis serves as contextual support rather than as the primary basis for biological conclusions. Building on this overview, the present review focuses on the structural classification, evolutionary diversification, phylogenetic relationships, and regulatory functions of MYB transcription factors in algae, with particular attention to their roles in growth, metabolism, and stress responses.

## 2. Overview of Research Trends and Literature Landscape of Algal MYB Transcription Factors

### 2.1. Data Sources and Methods

**Figure 1:**
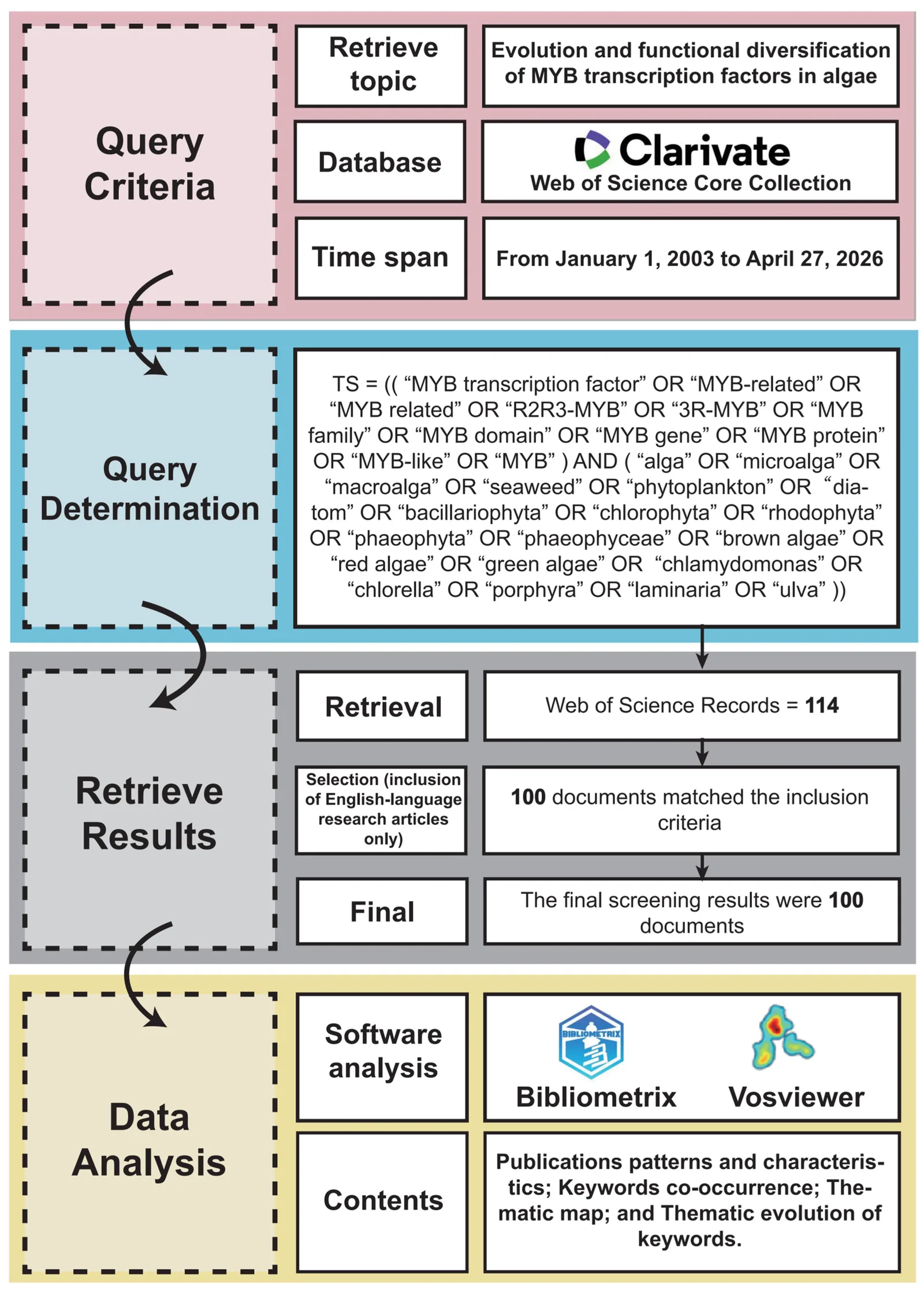
Flowchart of the literature search strategy on the evolution and functional diversification of MYB TFs in algae.

This study uses bibliometric methods to explore the research progress of MYB TFs in algae [24]. Web of Science (WoS) was the first broad-scope international bibliographic database. Therefore, over time it became the most influential bibliographic data source traditionally used for journal selection, research evaluation, bibliometric analyses, and other tasks [25]. The literature search is based on the Web of Science Core Collection (WOSCC) database, which is widely used in the field of life sciences as a comprehensive and authoritative citation index [26]. Because the bibliometric analysis relied exclusively on the Web of Science Core Collection, it may underestimate algal MYB research published in journals not indexed by this database. The search period is from 2003 to 2026, with the specific search date being 27 April 2026. This search strategy utilizes Boolean operators (are words and symbols, such as AND or NOT) to identify relevant literature focusing on MYB TFs and algae (including microalgae and macroalgae) [27]. By searching through the keywords in the Query Determination section of Figure 1, a total of 114 entries were obtained. After selection (including English language research articles) screening, 100 eligible documents were obtained, which will be used for subsequent bibliometric research. 

Keyword co-occurrence analysis was conducted using VOSviewer (version 1.6.20) to provide a descriptive overview of the major research themes in algal MYB transcription-factor studies. Keywords were included only when they occurred in at least six publications in the final dataset. This threshold retained 27 keywords for network construction. Co-occurrence link strengths were normalized using the association-strength method. The network was mapped using the default VOSviewer layout settings (attraction = 2 and repulsion = 0) and clustered using the VOS clustering technique with a resolution parameter of 1.00, a minimum cluster size of 1, and merging of small clusters enabled. No additional similarity or link-strength threshold was applied after the minimum-occurrence filter. The final network contained 27 items, 245 links, a total link strength of 483, and three keyword clusters. The thematic labels assigned to the clusters were determined manually after inspection of the most frequent and strongly connected keywords within each cluster and of the titles and abstracts of the associated publications. These labels were used only to summarize the dominant topics represented in the literature and do not constitute direct functional classification or experimental evidence for MYB transcription-factor activity [28].

### 2.2. Publication Trends and Research Landscape

**Figure 2:**
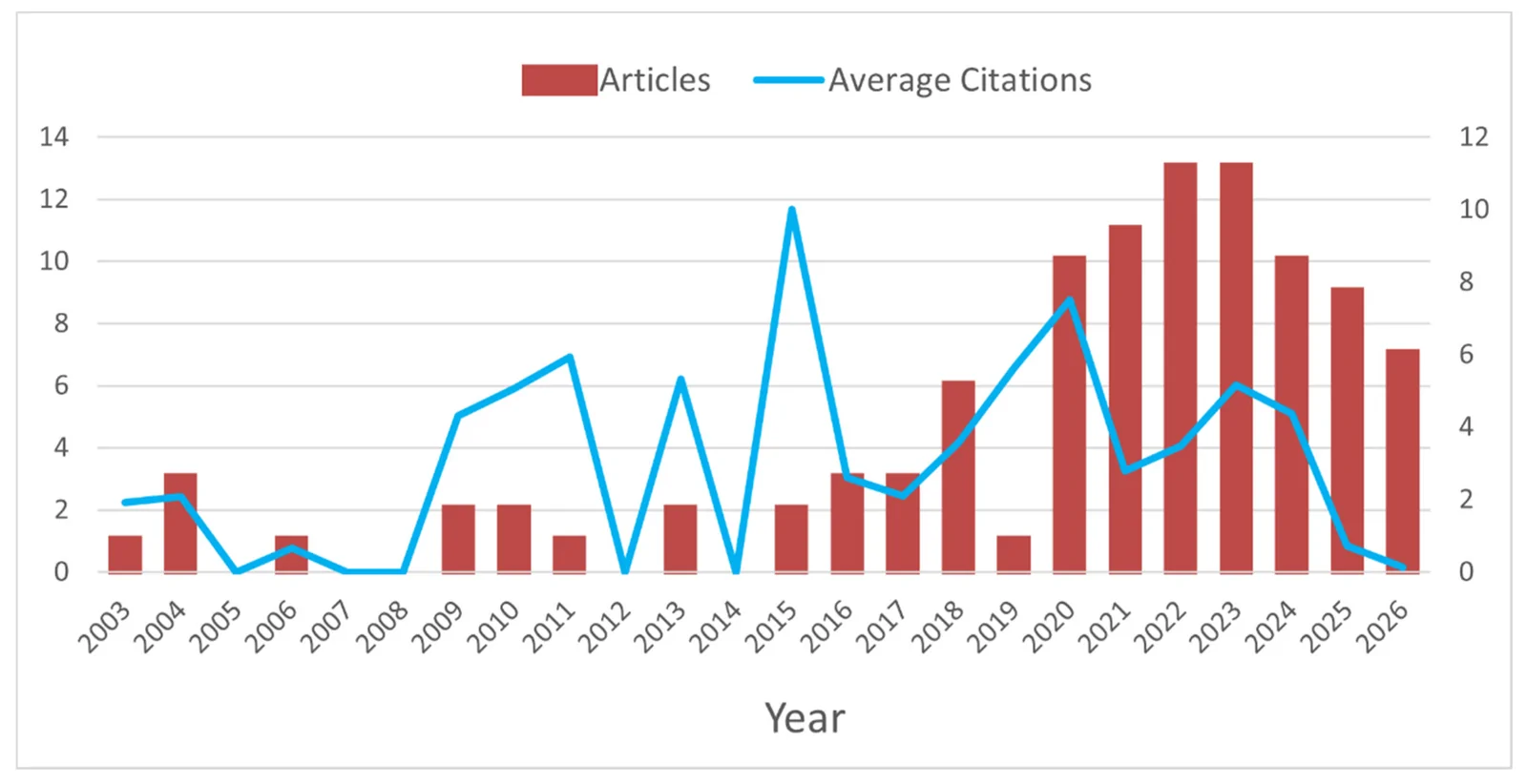
Annual publication output and average citations per article for studies on algal MYB transcription factors retrieved from the Web of Science Core Collection from 1 January 2003 to 27 April 2026. Bars represent the number of articles published each year (left *y*-axis), and the line represents the average citations per article (right *y*-axis). The 2026 value includes records indexed only up to 27 April 2026.

Annual publications on algal MYB transcription factors generally remained at three or fewer from 2003 to 2017, then increased to a peak of 13 in both 2022 and 2023 (Figure 2). The 2026 count is incomplete because the search covered publications only through 27 April 2026. The literature landscape further indicates that progress in algal MYB research has not been uniform across research questions. Existing studies have generated increasing information on MYB gene identification [29], family composition [30], and expression responses [31], whereas experimentally supported evidence for MYB function remains available for only a limited number of algal species and biological processes [32,33]. Consequently, the relationships among MYB structural classification, evolutionary diversification, and functional specialization are still not fully resolved. Therefore, the integration of these publications reveals the trend of MYB research in algae.

### 2.3. Major Research Themes

**Figure 3:**
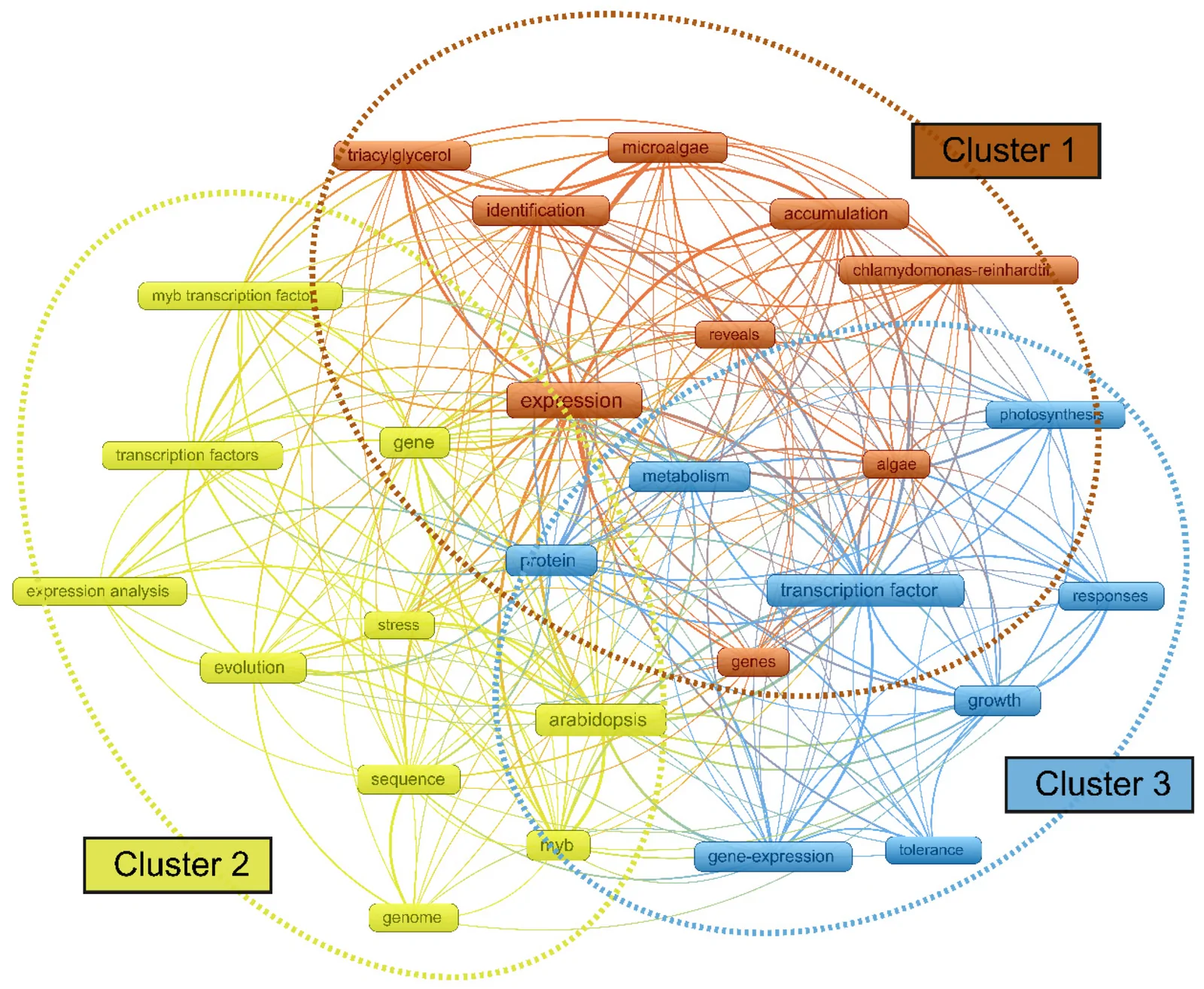
Keyword co-occurrence network of research on algal MYB transcription factors. The topic tags are summarized by the author based on keywords and are only used for descriptive identification. Keywords were extracted from Web of Science Core Collection records and included only if they appeared in at least six publications, yielding 27 keywords. Co-occurrence links were normalized using the association-strength method. The network was generated with VOSviewer (version 1.6.20) using the default layout settings (attraction = 2, repulsion = 0) and clustered with the VOS clustering technique (resolution = 1.00, minimum cluster size = 1, small cluster merging enabled). No additional similarity or link-strength thresholds were applied. The final network comprises 27 nodes, 245 links, and a total link strength of 483, forming three distinct clusters. Node size reflects keyword occurrence frequency; link thickness indicates co-occurrence strength; node colors denote algorithmically assigned clusters.

To identify the major topic tags represented in the literature, keyword co-occurrence analysis was performed using VOSviewer (version 1.6.20) [27]. (Figure 3). Given the limited size of the literature corpus and the relatively sparse keyword co-occurrence network, the resulting clusters and author-assigned topic tags were treated as descriptive summaries rather than stable or validated thematic findings.

Cluster 1 (brown) was characterized by terms including ‘microalgae’, ‘triacylglycerol’, ‘accumulation’, ‘identification’, ‘expression’, and ‘*Chlamydomonas reinhardtii*’. This cluster was therefore descriptively labeled as MYB identification and lipid-associated research in microalgae [34,35]. Cluster 2 (yellow) included ‘MYB transcription factor’, ‘transcription factors’, ‘expression analysis’, ‘evolution’, ‘sequence’, ‘genome’, and ‘stress’, and was labelled as MYB gene identification, evolutionary analysis, and expression profiling [36]. Cluster 3 (blue) contained terms related to ‘transcription factor’, ‘metabolism’, ‘photosynthesis’, ‘growth’, ‘responses’, ‘tolerance’, and ‘gene expression’. This cluster was labelled as transcriptional regulation of growth, metabolism, and environmental responses [37]. Among these, the green alga *C. reinhardtii* is the most commonly used model organism; it links multiple research keywords, indicating its significant role in current experimental research [38].

Collectively, the three clusters provide a thematic framework for the following sections, which examine MYB structural classification and evolutionary diversification, followed by experimentally supported functional roles in algal growth, metabolism, and environmental adaptation.

## 3. Structural Features and Evolutionary Patterns of Algal MYB Transcription Factors

### 3.1. Structural Classification of Algal MYB Transcription Factors

MYB TFs possess a highly conserved N-terminal DNA-binding domain consisting of 1–4 incomplete repeats (R) [39]. Each repeat contains 50–53 amino acids forming three α-helices, where the second and third helices adopt a helix-turn-helix (HTH) fold stabilized by three evenly spaced tryptophan residues that form a hydrophobic core [40].

Although MYB proteins have been identified in diverse algal lineages, no experimentally resolved 3-D structure of an algal MYB protein, either alone or in complex with DNA, is currently available to the best of our knowledge. However, modeled 3-D structures of algal MYB DNA-binding domains (DBD) containing one, two, or three MYB repeats provide preliminary structural representations of the corresponding MYB classes. The structure of the MYB DBD of brown algae was inferred through homology modeling based on the most similar sequence to non-plant MYB [18]. Consequently, the structural basis of DNA recognition by algal MYB proteins is inferred primarily from homologous MYB proteins characterized in land plants. The classic domain of the MYB family (PF00249) and its AlphaFold-predicted 3D structure are shown in Figure S1 [41]. In these proteins, the MYB DNA-binding domain consists of one or more repeats, each typically forming a three-helix bundle, with the recognition helix involved in DNA binding [42]. The absence of experimentally resolved algal MYB structures represents an important knowledge gap for future studies.

**Figure 4:**
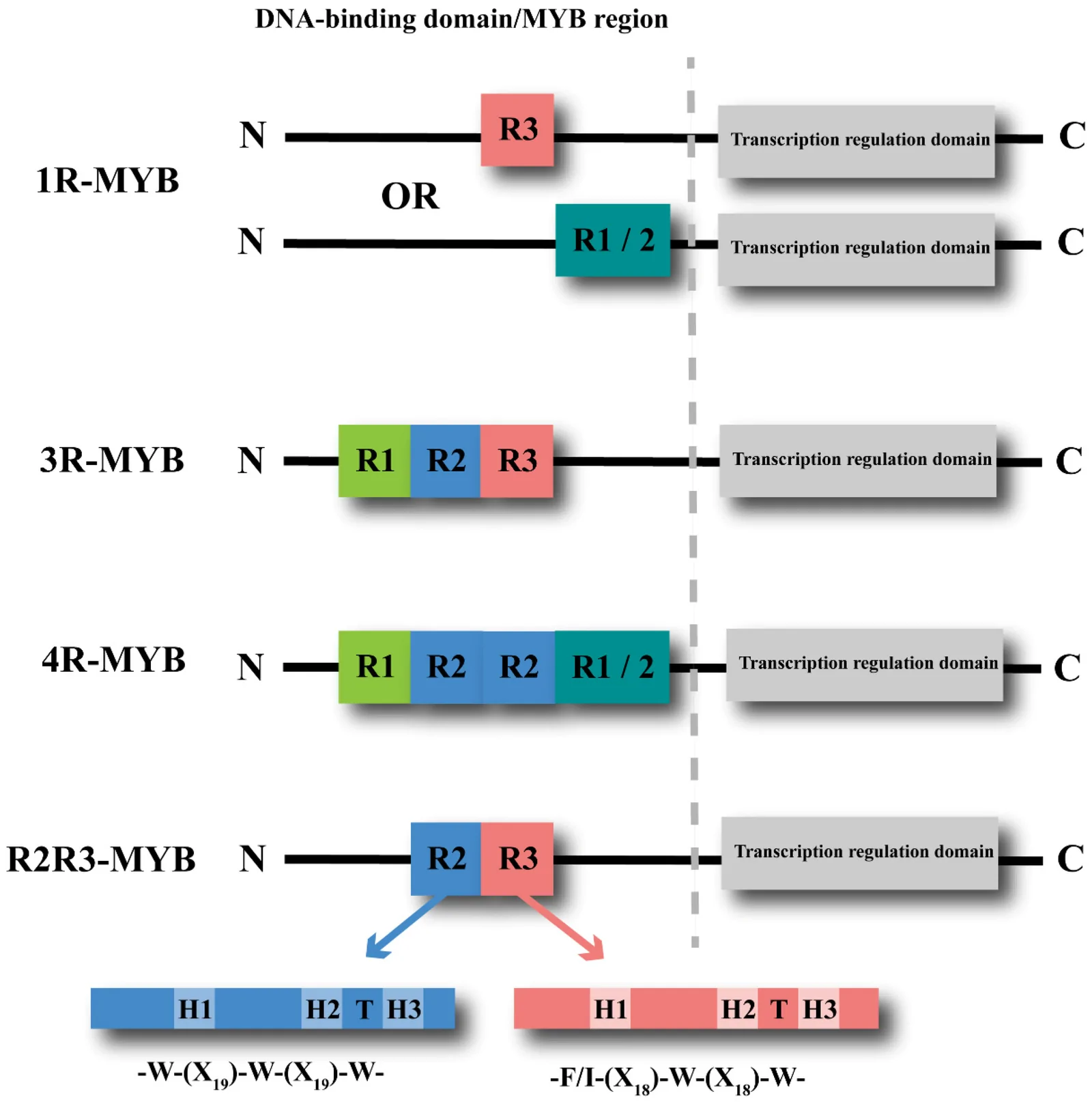
Schematic diagram of the protein structures of different MYB TFs. MYB TFs are divided into four groups based on the number of MYB domains. Each repeat sequence contains 50–53 amino acids, with three conserved tryptophan residues separated by 18–19 amino acid spacer sequences, forming a helix-turn-helix (HTH) domain [17,18,19,20,21,22,23,24,25,26,27,28,29,30,31,32,33,34,35,36,37,38,39,40,41,42,43,44].

MYB proteins are classified into four major classes based on repeat number and arrangement: 1R-MYB, 2R-MYB (R1R2), 3R-MYB (R1R2R3), and 4R-MYB (Figure 4) [17]. 2R-MYBs represent the largest MYB subgroup and are classified into 28 subgroups according to conserved C-terminal motifs [43,44]. They likely originated from 3R-MYBs through R1 repeat loss or duplication events [45] and mainly regulate metabolism, morphogenesis and stress responses in plants [46,47]. MYB-related proteins contain single or partial MYB repeats and function in morphogenesis, chloroplast development, secondary metabolism and phosphate starvation responses [48]. 3R-MYBs are evolutionarily conserved across eukaryotes but occur in low copy numbers in plants, where they regulate development and stress responses [49,50,51]. 4R-MYBs are highly conserved but remain poorly characterized in plants [52,53]. Additional 5R- and 6R-MYBs have also been reported in brown algae [18].

While MYB TFs have been extensively characterized in higher plants, with well-defined roles in metabolism, development, and stress responses [54], their functions in algae remain poorly understood. Recent genome-wide studies have identified and classified MYB gene families across diverse algal lineages (Table S1), in nearly 100 genera across eight major phyla, including model species such as *C. reinhardtii*, *Porphyridium purpureum*, *Nannochloropsis oceanica*, *Klebsormidium flaccidum*, *Saccharina japonica*, *Thalassiosira pseudonana*, *Vitrella brassicaformis*, and *Isochrysis galbana* [55]. The comparatively high number of MYB genes annotated in Galdieria may be associated with its adaptation to extreme environments, but this remains a hypothesis that requires testing with uniformly annotated, high-quality genome data.

All counts were obtained from published articles and the TAPscan v4 database [56], but the underlying genomes may differ in assembly completeness and gene annotation quality. These differences limit direct comparisons among species. Testing the proposed relationship between MYB abundance and adaptation will require a species-level comparison using genomes of comparable quality and a uniform annotation pipeline.

### 3.2. Evolutionary Characteristics of MYB in Algae

To investigate evolutionary relationships in algae, we focused on the 2R-MYB subfamily, which is the most extensively characterized group to date [57,58,59]. The phylogenetic tree was established according to the number of repetitions of the MYB domain. The protein sequence data are completely from the protein model of genome prediction. Comparative phylogenetic analysis was performed using MYB protein sequences from representative species across 8 algal phyla, together with sequences from three land plants: *Arabidopsis thaliana* (Angiosperm), *Marchantia polymorpha* (liverwort), and *Ginkgo biloba* (Gymnosperm). The land-plant sequences served as references for assessing the evolutionary relationships between algal and land-plant MYBs. For the 2R-MYB phylogenetic analysis, the candidate protein sequences were further re-evaluated based on the conserved MYB DNA-binding domain to improve sequence comparability and the reproducibility of the phylogenetic analysis. Protein sequences were searched against the Pfam MYB DNA-binding domain profile (PF00249; Myb_DNA-binding) using HMMER v3.4 [60]. Significant PF00249 domain matches were identified using the Pfam curated family-specific gathering threshold (--cut_ga) [61]. Proteins containing exactly two significant PF00249 hits were retained in the 2R-MYB dataset, whereas proteins containing one or three significant hits were excluded. For each retained protein, the two PF00249 hits were ordered according to their positions in the protein sequence and designated as the first (R1) and second (R2) MYB repeats. The amino acid regions corresponding to the HMMER alignment coordinates (ali_from to ali_to) of the two PF00249 hits were extracted and concatenated in the R1–R2 order for subsequent multiple sequence alignment and phylogenetic analysis. This domain-based procedure minimized the influence of highly variable non-MYB regions and long inter-repeat linker sequences on sequence alignment. The resulting phylogenetic tree was treated and visualized as an unrooted tree because no explicit outgroup was designated for rooting. The protein sequences used in this study mainly come from the TAPscan v4 database and protein sequences reported in published studies (Table S1). The reason for choosing this database is that it follows a unified transcription factor identification process and family classification rules, systematically annotating multiple algal species, which facilitates cross-species comparisons. The species included in the analysis were required to meet the following conditions: they had relatively complete and publicly accessible genomic or proteomic data; they had MYB or MYB-related family annotations in TAPscan v4; and their taxonomic status and species names could be cross-checked with public literature and mainstream classification databases.

**Figure 5:**
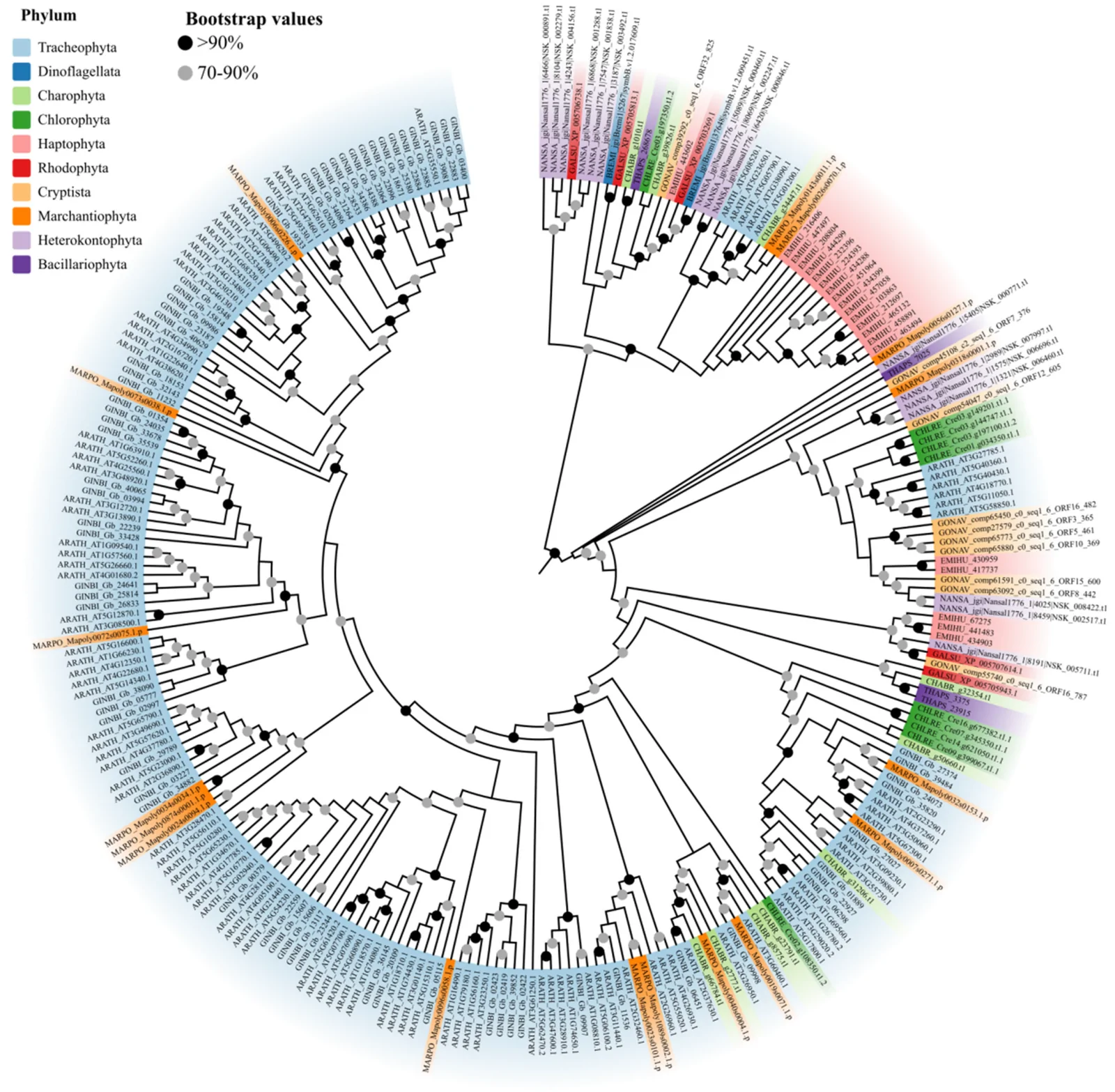
Phylogenetic tree of the 2R-MYB transcription factor family in algae. The tree was constructed using the maximum-likelihood method based on 2R-MYB protein sequences from 8 algal phyla, 1 angiosperm (Tracheophyta phylum), 1 gymnosperm (Tracheophyta phylum), and 1 liverwort (Marchantiophyta phylum). Multiple sequence alignment was performed with MAFFT (L-INS-i), trimal (v. 1.4.rev15)—gt 0.3—st 0.001 [64] for construction purposes and phylogenetic reconstruction was conducted using IQ-TREE3 [65] with the maximum likelihood method [66]. The best-fit substitution model and rate heterogeneity were automatically determined by ModelFinder (Auto) with ML-optimized state frequencies [67]. Branch support was assessed using 1000 Ultrafast Bootstrap (UFBoot2) [68] replicates and 1000 SH-aLRT replicates [69]. Numbers at the nodes represent SH-aLRT (%) and UFBoot2 (%) support values, respectively. The tree was visualized and optimized using TVBOT [70]. Different colors and clades represent major algal phyla.

The 2R-MYB phylogeny shows a heterogeneous distribution of 268 sequences across the sampled taxa (Figure 5). Land-plant sequences, particularly those from *Arabidopsis thaliana*, *Ginkgo biloba* and *Marchantia polymorpha*, are heavily clustered in the phylogenetic tree (blue plate), while many algal sequences are only clustered in a small part. It shows algae generally contain fewer 2R-MYB members than land plants [62]. The groups are not completely separated: several *Chara braunii* sequences (light green block) occur alongside land-plant sequences. Charophytes exhibit greater similarity to land plants in gene structure and domain organization [57]. And *C. reinhardtii* sequences are distributed across multiple branches rather than forming a single monophyletic clade. The occurrence of chlorophyte and charophyte sequences among land-plant homologs is consistent with the presence of 2R-MYB lineages before terrestrialization, as supported by broader comparative analyses [8]. Subsequent duplication has contributed to the expansion of 2R-MYBs in land plants [63]. Because several deeper relationships have limited bootstrap support, the present tree alone cannot resolve the timing of these events or establish specific instances of gene loss or horizontal gene transfer.

The 255 1R-MYB (MYB-related) sequences from the sampled taxa are distributed across multiple branches of the phylogenetic tree (Figure S2). Several *C. reinhardtii* sequences occur close together, while others appear on separate branches. Sequences from *Emiliania huxleyi* and *Galdieria sulphuraria* also form enriched groups, with bootstrap support above 90% for several internal nodes. Sequences from different algal lineages occur near one another elsewhere in the tree. Overall, the phylogeny shows both supported local clustering and broader interspersion of MYB-related sequences. Because some deeper nodes lack strong bootstrap support, relationships among the broader groups remain uncertain.

Owing to the restricted taxonomic distribution and low copy number of 3R-MYB genes, relatively few informative sequences were available for phylogenetic reconstruction (Figure S3). Several internal nodes have low bootstrap support, limiting the evolutionary relationships that can be inferred from these trees. Restricted sampling and limited phylogenetic signal in the aligned MYB DNA-binding regions may contribute to this uncertainty. Relationships at weakly supported nodes are treated as unresolved, without assigning specific gene duplication or loss events to them.

The 4R-MYB dataset comprised nine sequences. The inferred topology was treated as exploratory, and no firm conclusions regarding relationships among algal lineages were drawn in the absence of branch support values.

## 4. The Function of MYB Transcription Factors in Algae

**Figure 6:**
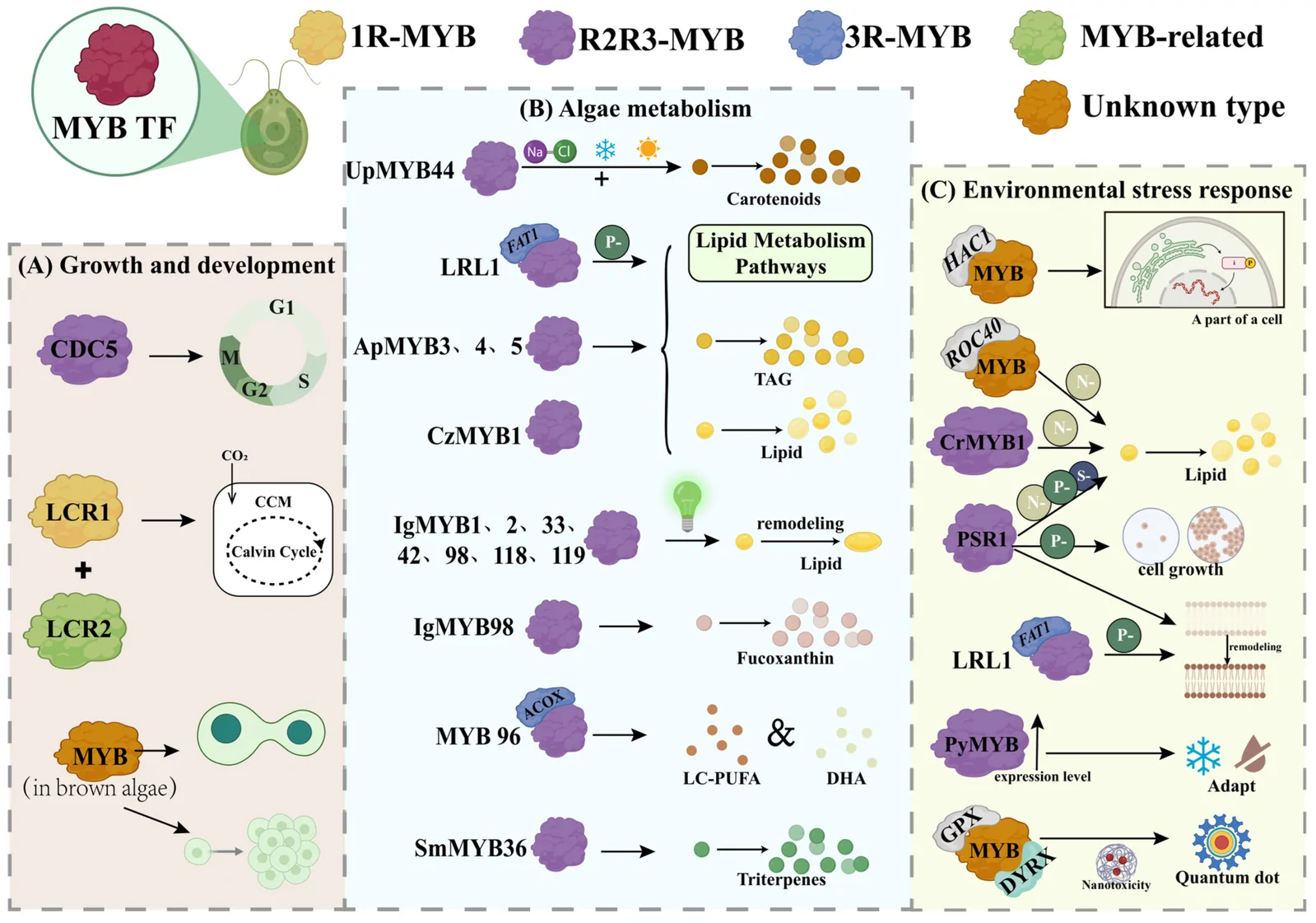
MYBTFs function as key regulators in diverse biological processes in algae [32,33]. MYBTFs, as a key class of regulatory proteins in algae, extensively participate in and integrate core biological processes such as growth and development, material metabolism, and environmental stress responses. They serve as crucial molecular hubs enabling algae to adapt to their environment and achieve efficient production. (**A**) MYB TFs regulate algal growth and development, including cell cycle, CO_2_ concentration mechanism (CCM), and multicellular development. (**B**) MYB TFs modulate primary and secondary metabolism, including lipid/TAG biosynthesis, carotenoid production, LC-PUFA/DHA synthesis, and terpenoid pathways. (**C**) MYB TFs mediate environmental stress responses, including nutrient starvation (N, P), abiotic stresses, and biotic/nanotoxicity adaptation [22,71].

MYB TFs are central regulators of algal growth, development, metabolism and stress responses, acting as key integrators of cellular functions via transcriptional control across species (Figure 6; Table S2). They govern cell cycle progression and carbon acquisition [31,71]. In *C. reinhardtii*, the MYB-related CDC5 is essential for cell cycle progression, consistent with the conserved role of MYB proteins in eukaryotic cell division [21]. LCR1 regulates the CO_2_-responsive *Cah1* gene encoding a carbonic anhydrase critical for the carbon concentrating mechanism (CCM), linking MYB activity to photosynthetic efficiency under fluctuating CO_2_ [72,73]; its co-regulator LCR2 further modulates CCM-associated transcriptional networks [74]. Brown algae show expanded 3R-MYB subfamilies regulating cell division, differentiation and development, while *Chlorella vulgaris* 3R-MYB proteins control the G2/M transition [75].

MYB TFs govern algal lipid biosynthesis and secondary metabolite production [76]. In *Chromochloris zofingiensis*, the 2R-MYB CzMYB1 is a global regulator of lipid metabolism, controlling triacylglycerol (TAG) biosynthesis and accumulation [33]. In *C. reinhardtii*, LRL1 is involved in phosphorus depletion responses [77], while MYB factors regulate the MEP pathway [78]. In *Auxenochlorella protothecoides*, ApMYB3–5 may play a potential role in regulating lipid synthesis and TAG production through gene expression in lipid metabolism [22]. Beyond lipids, *Ulva prolifera* UpMYB44 positively regulates carotenoid biosynthesis by activating key pathway genes [79], whereas *Isochrysis galbana* IgMYB98 potentially negatively regulates fucoxanthin biosynthesis during light-induced metabolic remodeling [80]. MYB proteins also cooperate with other transcription factors to regulate long-chain polyunsaturated fatty acid (LC-PUFA) synthesis [81]. In *Nannochloropsis oceanica*, MYB is the most diverse and active transcription factor family that responds to heat stress conditions [82].

MYB TFs also mediate algal adaptation to nutrient limitation and abiotic stress. The MYB-like PSR1 is functionally conserved across algae under phosphorus starvation: in *C. reinhardtii*, it activates phosphorus uptake and metabolism genes, enhancing deficiency tolerance [83,84,85,86]; in *P. tricornutum*, PtPSR regulates phosphorus acquisition and lipid remodeling by directly binding target gene promoters [87]; and in *N. oceanica*, PSR1 drives membrane lipid remodeling via non-phosphorus lipid synthesis [23]. Under nitrogen deprivation, *C. reinhardtii* MYB1 promotes lipid accumulation by facilitating fatty acid transport [88], and ROC40 regulates TAG accumulation [89]. Additionally, *Chlamydomonas’* MYB genes are rapidly induced under salt stress to regulate osmotic balance and antioxidant pathways [90]; in *Schizochytrium* sp., MYB15 mediates docosahexaenoic acid biosynthesis and salt tolerance [16]. *P. tricornutum* MYB TFs (LRM) modulate light-harvesting complex gene expression in response to light intensity changes [91].

## 5. Conclusions 

This review highlights that algal MYB transcription factors comprise structurally diverse families that have undergone lineage-specific diversification across the major algal groups. Available phylogenetic and comparative evidence provides an initial framework for understanding the evolutionary relationships of algal MYB proteins. Current functional evidence supports roles for algal MYB transcription factors in the regulation of growth, metabolism, and environmental responses, but these functions have been experimentally characterized in only a limited number of species and genes.

## 6. Future Perspectives

Future research should prioritize standardized genome-based annotation of MYB repertoires across phylogenetically diverse algae, together with systematic functional validation of representative MYB genes. Combining targeted genetic perturbation with transcriptomic, chromatin-level, and metabolomic analyses will be essential to identify MYB target genes and define conserved versus lineage-specific regulatory networks. In addition, the absence of experimentally resolved algal MYB–DNA structures remains an important knowledge gap. Addressing these questions will advance the understanding of MYB evolution and regulatory function in algae and provide a stronger basis for their future application in algal biotechnology.

## Data Availability

The data presented in this study are openly available in databases such as Web of science, Scopus, PubMed, Google Scholar and TAPscan v4 (Git-Hub). The datasets generated during the current study are available in the Figshare repository, https://doi.org/10.6084/m9.figshare.34184421. The remaining data discussed in this review article are obtained from the published studies cited within this manuscript. Complete details of these sources are provided in the reference list.

## Supplementary Materials

The following supporting information can be downloaded at: https://www.mdpi.com/article/10.3390/biology15201865/s1, Figure S1: AlphaFold-predicted structural model of a MYB-like DNA-binding domain (PF00249); Table S1: Distribution of MYB family subtypes (1R-6R and MYB-related) across different algal lineages [92,93]; Figure S2: Phylogenetic trees of 1R-MYB family; Figure S3: Phylogenetic trees of 3R-MYB family; Table S2. Experimentally characterized MYB transcription factors in algae.
