# Supplementary material for: Evolutionary Diversification and Functional Roles of MYB Transcription Factors in Algae: Insights from Genomic Distribution and Regulatory Networks
Source: Biology (Basel). 2026 Oct 16;15(20):1865. doi: 10.3390/biology15201865 (PMC13701856; doi:10.3390/biology15201865)
Supplement: Supplementary file 1 [file biology-15-01865-s001.zip › biology-4583430-supplementary.pdf]

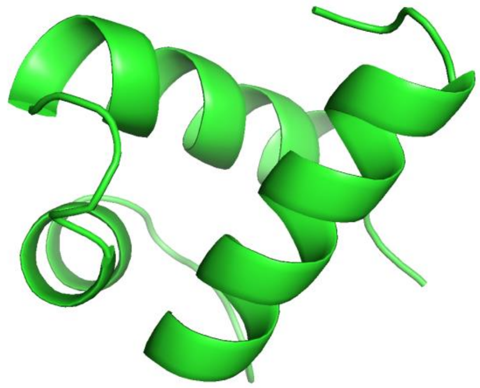

Figure S1. AlphaFold-predicted structural model of a MYB-like DNA-binding domain (PF00249).
The model represents the PF00249 containing MYB-like DNA-binding domain of protein A0AAD7W3P5 from Aldrovanda affinis, as displayed in InterPro/AlphaFold. The model illustrates the predicted three-helix architecture characteristic of a MYB repeat. Colours indicate AlphaFold local-confidence scores (pLDDT). This model is a computational prediction from a land-plant protein and is included solely as a structural reference; it does not represent an experimentally resolved or algal MYB structure [41].

Table S1. Distribution of MYB family subtypes (1R-6R and MYB-related) across different algal lineages. Data were compiled from TAPscan v4 [56] and peer-reviewed genome-wide MYB or transcription-factor studies. Values obtained represent database-predicted MYB candidates based on the reference genome/proteome annotation adopted by the database. Values obtained from published studies represent the classifications or MYB counts reported in the corresponding original articles. For species in which the original study did not resolve MYB proteins into individual repeat-number classes, the reported MYB count is displayed as a merged entry across the repeat-class columns. "/" indicates that the corresponding information was not reported or was unavailable in the cited source.

Phylum	Genus	2R	3R	4R	1R&related	5/6R	Reference
Bacillariophyta	*Cyclotella*	8	6	1	18		TAPscan v4
	Fistulifera	10	8	0	20		
	Fragilariopsis	18	8	0	18		
	Minidiscus	15	4	0	46		
	Phaeodactylum	9	5	2	14	1	[18]
	Pseudo-nitzschia	10	3	0	9		TAPscan v4
	Seminavis	7	4	1	14		
	Thalassiosira	22	6	2	35		
Charophyta	*Chara*	10	2	1	12		
	Chaetosphaeridium	0	0	6	5		
	Chlorokybus	3	0	0	11		
	Coleochaete	24	10	3	35		
	Closterium	39	12	0	70		
	Entransia	15	4	1	36		
	Klebsormidium	47	4	0	29		
	Mesotaenium	14	2	1	26		
	Mougeotia	37	8	0	97		
	Nitella	13	4	8	61		
	Penium	23	0	0	39		
	Spirogloea	18	8	0	35		
	Spirogyra	93	5	0	110		
	Zygnema	112	5	6	167		
Chlorophyta	*Asterochloris*	10	2	0	15		
	Auxenochlorella	6	1	0	14		[22]
	Bathycoccus	9	2	0	11		TAPscan v4
	Botryococcus	8	5	0	16		
	Caulerpa	5	0	0	8		
	Chlamydomonas	55	4	2	106		
	Chloropicon	6	1	1	20		
	Chlorella	27	4	0	45		
	Chromochloris	17	0	0	18		[33]
	Coccomyxa	7	1	1	14		TAPscan v4
	Dunaliella	8	0	0	12		

	Edaphochlamys	13	1	0	42		
	Enallax	17	3	0	17		
	Flechtneria	19	2	0	15		
	Gonium	12	0	0	13		
	Micractinium	12	2	0	14		
	Ostreococcus	27	5	0	49		
	Picocystis	1	0	0	5		
	Picochlorum	13	4	0	25		
	Raphidocelis	22	3	0	15		
	Symbiochloris	10	2	0	21		
	Tetradesmus	38	9	0	31		
	Tetrabaena	9	0	0	15		
	Tetraselmis	12	2	0	32		
	Trebouxia	6	3	0	12		
	Ulva	4	1	0	9		
	Volvox	17	1	1	20		
	Monoraphidium	13	0	0	20		
Cryptista	*Cryptomonas*	14	0	0	18		
	Goniomonas	11	4	1	47		
	Guillardia	18	3	0	71		
Dinoflagellata	*Amoebophrya*	1	0	0	1		
	Breviolum	2	0	0	4		
	Cladocopium	0	0	0	3		
	Fugacium	1	0	0	1		
	Symbiodinium	2	0	0	4	1	[18]
Haptophyta	*Emiliania huxleyi*	46	3	1	56		[93]
	Phaeocystis	139	1	0	68		[55]
	Chrysochromulina	63	2	0	54		
	Isochrysis	22	6	3	19		[92]
Heterokontophyta	*Aureococcus*	20	8	1	21		TAPscan v4
	Ascophyllum	12	10	0	16		
	Chordaria	12	11	1	16		
	Cladosiphon	16	16	0	41	5	[18]
	Chrysoparadoxa	10	9	1	12		TAPscan v4
	Desmarestia	11	11	0	13		
	Dictyota	11	6	0	15		
	Discosporangium	10	6	1	22		
	Ectocarpus	49	47	1	109	3	[18]
	Fucus	12	8	0	14		TAPscan v4
	Heterosigma	3	1	0	8		
	Nannochloropsis	46	18	1	63		
	Nemacystus	14	14	0	42	4	[18]
	Ochromonas	25	10	0	39		TAPscan v4
	Paraphysomonas	9	7	3	19		
	Pleurocladia	9	6	1	12		
	Porterinema	10	9	0	20		
	Pylaiella	13	9	0	23		

	Saccharina	21	17	0	27	1	[18]
	Sargassum	16	9	0	20		TAPscan v4
	Schizocladia	12	8	0	16		
	Scytosiphon	14	10	0	19		
	Tribonema	31	12	0	21		
	Undaria	16	3	0	13		
Rhodophyta	*Calliarthron*	6	1	1	8		
	Chondrus	11			15		[55]
	Cyanidium	1	1	0	12		TAPscan v4
	Galdieria	68	22	10	219		
	Gracilariopsis	7	2	0	24		
	Porphyridium	38	1	0	78		
	Porphyra	10	0	0	29		
	Cyanidioschyzon	17			18		[55]

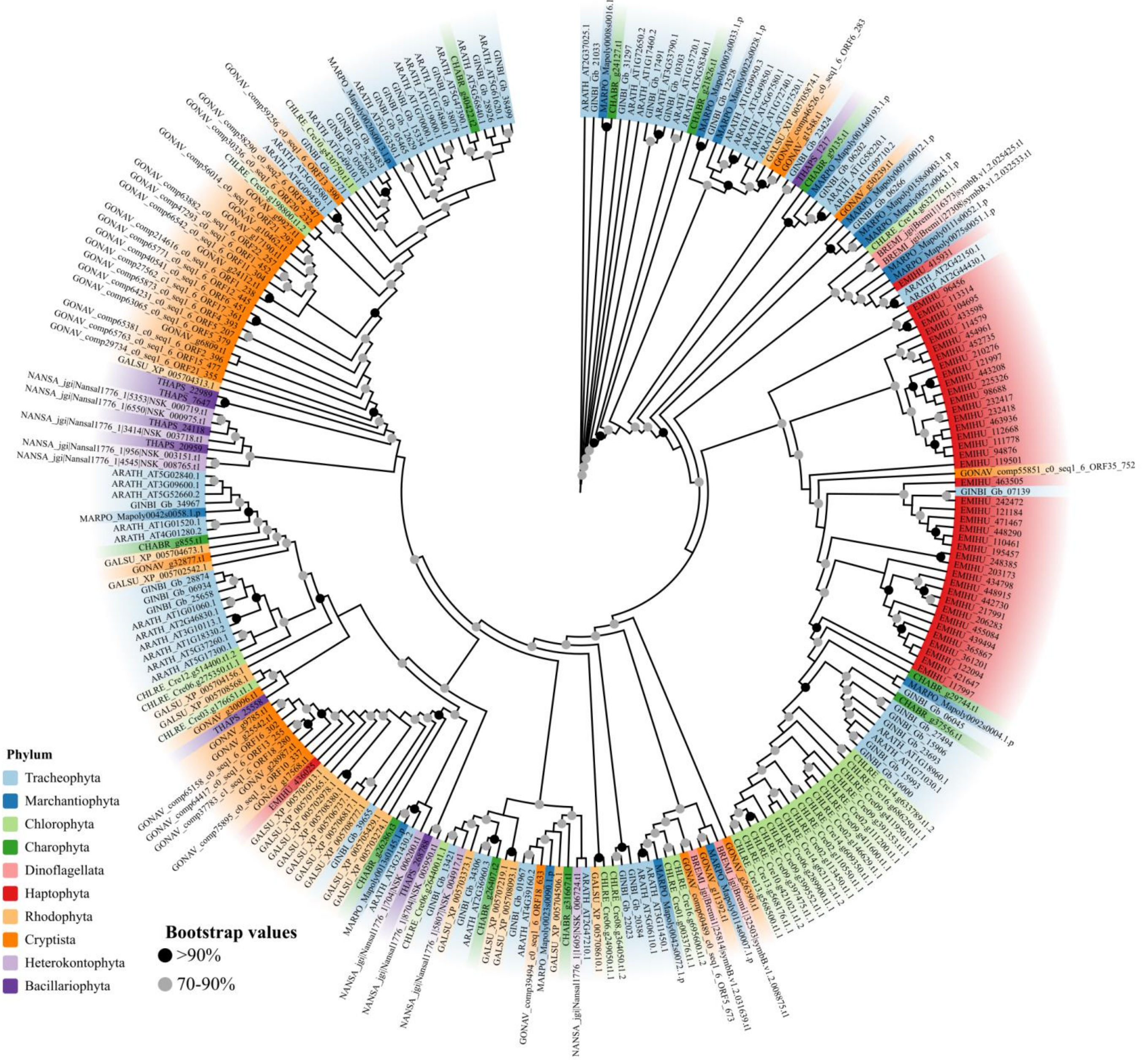

Figure S2. Phylogenetic trees of 1R-MYB family. Based on 8 algal phyla, 1 angiosperm (Tracheophyta phylum), 1 gymnosperm (Tracheophyta phylum), and 1 liverwort (Marchantiophyta phylum). Constructed using MAFFT (L-INS-i) alignment, trimal (v. 1.4.rev15)—gt 0.3—st 0.001 for construction purposes and IQ-TREE3 with the maximum likelihood method [66]. The best-fit substitution model and rate heterogeneity were automatically determined by ModelFinder (Auto) with ML-optimized state frequencies [67]. Branch support was assessed using 1000 Ultrafast Bootstrap (UFBoot2) [68] replicates and 1000 SH-aLRT replicates [69]. The phylogenetic tree has been enhanced using TVBOT [70].

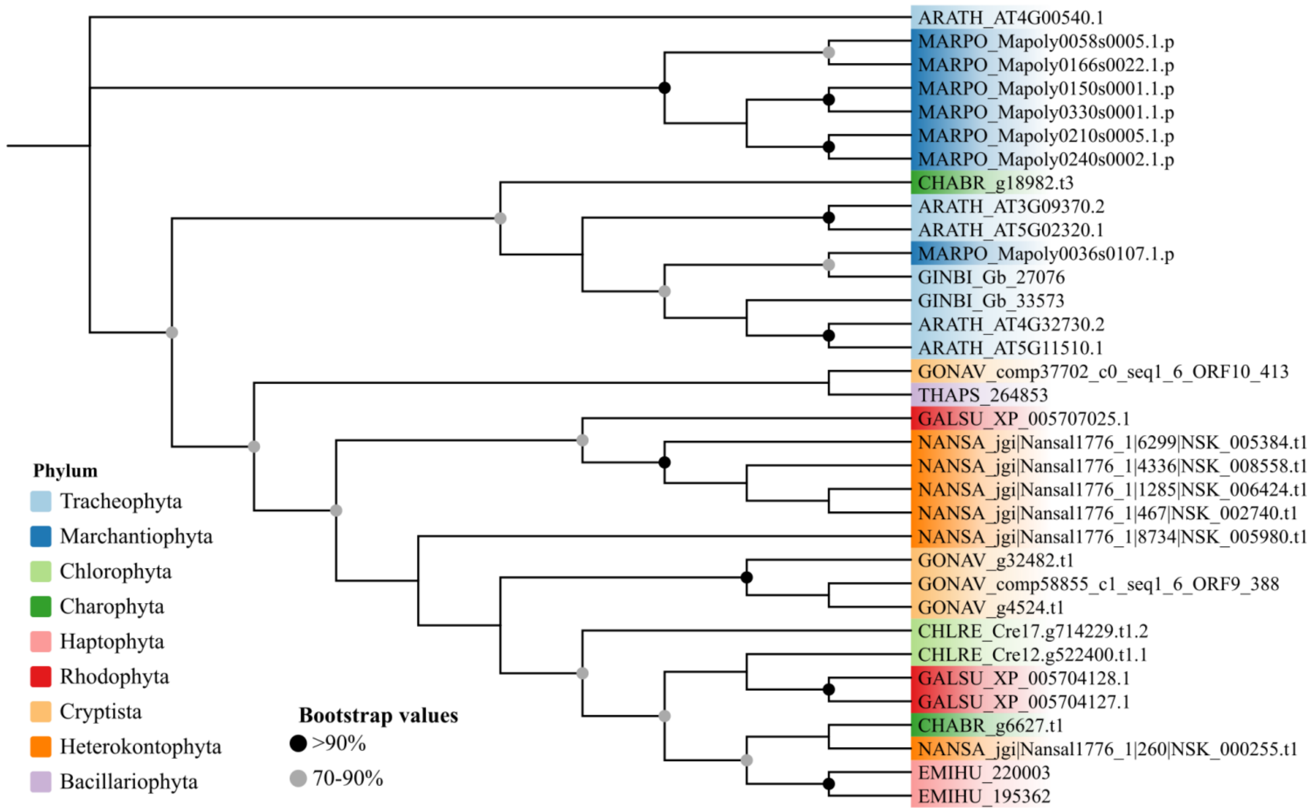

Figure S3. Phylogenetic trees of 3R-MYB family. Based on 7 algal phyla (Dinoflagellata doesn't have the 3R sequence), 1 angiosperm (Tracheophyta phylum), 1 gymnosperm (Tracheophyta phylum), and 1 liverwort (Marchantiophyta phylum). Constructed using MAFFT (L-INS-i) alignment, trimal (v. 1.4.rev15)—gt 0.3—st 0.001 for construction purposes and IQ-TREE3 with the maximum likelihood method [66]. The best-fit substitution model and rate heterogeneity were automatically determined by ModelFinder (Auto) with ML-optimized state frequencies [67]. Branch support was assessed using 1000 Ultrafast Bootstrap (UFBoot2) [68] replicates and 1000 SH-aLRT replicates [69].The phylogenetic tree has been enhanced using TVBOT [70].

Table S2. Experimentally characterized MYB transcription factors in algae

Species	*Gene name*	MYB type	Functional verification method	Phenotype	Reference
Chlamydomonas reinhardtii	*CrCDC5*	MYB-related	Insertional mutants (crcdc5-1/2); flow cytometry; starch/lipid quantification	Prolonged S/M phase, abnormal cell division, excessive accumulation of starch and lipids	[21]
Chlamydomonas reinhardtii	*CrLCR1*	MYB-related	Gene expression analysis; promoter binding assay (EMSA)	Regulates expression of the CO_2-responsive gene Cah1, affecting the carbon concentrating mechanism (CCM)	[72]
Chromochloris zofingiensis	*CzMYB1*	2R-MYB	Insertional mutagenesis; Y1H; EMSA; transcriptome analysis	Global regulator of TAG biosynthesis and accumulation; TAG significantly decreased upon disruption	[33]
Chlamydomonas reinhardtii	*CrMYB1*	2R-MYB	Insertional mutant (crmyb1-1); overexpression; transcriptome analysis	Positively regulates TAG accumulation under nitrogen starvation; mutant shows up to 66% reduction in TAG	[32]
Chlamydomonas reinhardtii	*CrLRL1*	MYB-related	Mutant (lrl1-1); metabolome analysis	Involved in lipid remodeling and secondary metabolism regulation under phosphorus starvation	[77]
Chlamydomonas reinhardtii	*CrROC40*	MYB-related	Mutant (roc40); gravimetric lipid quantification	Impaired TAG accumulation under nitrogen starvation; positively regulates DGTT1 transcription	[89]
Phaeodactylum tricornutum	*PtPSR*	MYB-like (related)	CRISPR gene editing (ptpsr mutant); EMSA; transcriptome + lipidome	Regulates phosphorus acquisition and membrane lipid remodeling; mutant impaired in phosphorus starvation response	[87]
Nannochloropsis oceanica	*NoPSR1*	MYB-like (related)	Gene knockout (nopsr1); lipidome analysis	Essential factor for membrane lipid remodeling under phosphorus starvation; severely impaired membrane lipid dynamics upon loss	[23]

Ulva prolifera	*UpMYB44*	2R-MYB	Y1H; EMSA; overexpression; stress treatment	Positively regulates carotenoid biosynthesis; directly binds UpPDS promoter	[79]
Schizochytrium sp.	*MYB15*	3R-MYB	Gene knockout (ΔMYB15); EMSA; DNase I footprinting; transcriptome	DHA and total lipids increased by 46.7% and 27.1%, respectively, upon deletion; enhanced salt tolerance	[16]
Phaeodactylum tricornutum	*LRM (LHC Regulating Myb)*	MYB-related	Antisense RNA interference (RNAi)	LRM knockdown strain impaired in photoacclimation; LHC gene expression no longer properly adjusts to light intensity; abnormal chlorophyll content changes	[91]
Chlamydomonas reinhardtii	SmMYB36 (heterologous)	/	Heterologous overexpression (SmMYB36 from *Salvia miltiorrhiza* transferred into *C. reinhardtii*)+qPCR	Proposed to regulate the MEP pathway	[78]
